# Distribution and Genomic Variation of Thermophilic Cyanobacteria in Diverse Microbial Mats at the Upper Temperature Limits of Photosynthesis

**DOI:** 10.1128/msystems.00317-22

**Published:** 2022-08-18

**Authors:** Eric D. Kees, Senthil K. Murugapiran, Annastacia C. Bennett, Trinity L. Hamilton

**Affiliations:** a Department of Plant and Microbial Biology, University of Minnesota, St. Paul, Minnesota, USA; b Biotechnology Institute, University of Minnesota, St. Paul, Minnesota, USA; UiT—The Arctic University of Norway

**Keywords:** cyanobacteria, *Synechococcus*, photosynthesis, metagenomes, metagenome assembled genomes, MAGs, microbial mats, hot springs, nitrogen fixation, ecotypes

## Abstract

Thermophilic cyanobacteria have been extensively studied in Yellowstone National Park (YNP) hot springs, particularly during decades of work on the thick laminated mats of Octopus and Mushroom springs. However, focused studies of cyanobacteria outside these two hot springs have been lacking, especially regarding how physical and chemical parameters along with community morphology influence the genomic makeup of these organisms. Here, we used a metagenomic approach to examine cyanobacteria existing at the upper temperature limit of photosynthesis. We examined 15 alkaline hot spring samples across six geographic areas of YNP, all with various physical and chemical parameters and community morphology. We recovered 22 metagenome-assembled genomes (MAGs) belonging to thermophilic cyanobacteria, notably an uncultured *Synechococcus*-like taxon recovered from a setting at the upper temperature limit of photosynthesis, 73°C, in addition to thermophilic *Gloeomargarita*. Furthermore, we found that three distinct groups of *Synechococcus*-like MAGs recovered from different temperature ranges vary in their genomic makeup. MAGs from the uncultured very-high-temperature (up to 73°C) *Synechococcus*-like taxon lack key nitrogen metabolism genes and have genes implicated in cellular stress responses that diverge from other *Synechococcus*-like MAGs. Across all parameters measured, temperature was the primary determinant of taxonomic makeup of recovered cyanobacterial MAGs. However, total Fe, community morphology, and biogeography played an additional role in the distribution and abundance of upper-temperature-limit-adapted *Synechococcus*-like MAGs. These findings expand our understanding of cyanobacterial diversity in YNP and provide a basis for interrogation of understudied thermophilic cyanobacteria.

**IMPORTANCE** Oxygenic photosynthesis arose early in microbial evolution—approximately 2.5 to 3.5 billion years ago—and entirely reshaped the biological makeup of Earth. However, despite the span of time in which photosynthesis has been refined, it is strictly limited to temperatures below 73°C, a barrier that many other biological processes have been able to overcome. Furthermore, photosynthesis at temperatures above 56°C is limited to circumneutral and alkaline pH. Hot springs in Yellowstone National Park (YNP), which have a large diversity in temperatures, pH, and geochemistry, provide a natural laboratory to study thermophilic microbial mats and the cyanobacteria within. While cyanobacteria in YNP microbial mats have been studied for decades, a vast majority of the work has focused on two springs within the same geyser basin, both containing similar community morphologies. Thus, the drivers of cyanobacterial adaptations to the upper limits of photosynthesis across a variety of environmental parameters have been understudied. Our findings provide new insights into the influence of these parameters on both taxonomic diversity and genomic content of cyanobacteria across a range of hot spring samples.

## INTRODUCTION

Oxygenic photosynthesis in *Cyanobacteria* is among the most impactful microbial innovations in Earth’s history and accounts for the largest source of O_2_ in the atmosphere. While photosynthesis has had approximately 2.5 to 3.5 billion years of evolution (1–3), it is constrained to temperatures of <73°C in alkaline environments and <56°C in acidic environments (4–8).

Characterizations of high-temperature cyanobacteria have been most extensively done in Hunter’s Hot Springs, Oregon, USA, and in thick laminated microbial mats of alkaline Mushroom Spring, Octopus Spring, and the mat and streamer communities in White Creek (9) in Yellowstone National Park (YNP), Wyoming, USA, hot springs. A limited number of studies have focused on phototrophic hot spring communities elsewhere in YNP (10–14). The most prevalent cyanobacteria found at or near the 73°C limit in these springs are those in a *Synechococcus*-like clade (15–17). Since these thermophilic *Synechococcus*-like cyanobacteria form a basal clade, distantly related to all other known *Synechococcus* species, they were recently classified under the proposed genus name *Leptococcus* (18, 19) or, more recently, *Thermostichus* (20). For the purposes of clarity and contextualization around existing literature, we refer to these cyanobacteria in the present study as belonging to the *Synechococcus-*like A/B lineage (10, 15, 17, 21–24).

Molecular probing of *Synechococcus*-like cyanobacteria in Octopus and Mushroom springs revealed several “ecotypes,” or sequence variants occupying different niche spaces defined by environmental variables such as temperature and light (15, 21, 22, 24–26). These ecotypes were organized into major categories designated “A” and “B.” “A” ecotypes (including the isolated and sequenced strain JA-3-3Ab, referred to here and elsewhere as OS-A) tend to occupy the highest temperatures up to 73°C, while “B” ecotypes [including JA-2-3B′a(2-13), or OS-B′] occupy a slightly lower range, up to 65°C (15, 17, 25). A and B ecotypes have been more finely delineated along temperature gradients into A, A′, A′′, B′ and so on, generally with each “prime” designation indicating a temperature range trending upward (15). Similar niche differentiation along temperature gradients was described for *Synechococcus*-like cyanobacteria in Hunter’s Hot Springs, and at present, the only cultured A′-like cyanobacteria were isolated from this location at 70°C (27–30). Biogeography plays an additional role in genetic divergence of ecotypes within A/B-lineage clades (10). In the present study, we were primarily interested in the genetic factors that drive ecological diversification among A/B-lineage populations, particularly in YNP hot springs with differing pH, temperature, and geochemistry.

While the underlying genetic factors driving adaptation to different temperatures among *Synechococcus*-like A/B-lineage organisms remain largely unresolved, some specific adaptations to the highest temperatures have been identified. For example, more thermostable variants of RuBisCO have been found to extend the thermal niche in an A/B-lineage clade from Hunter’s Hot Springs (30). Comparisons between OS-B′ (53 to 60°C) and OS-A (58 to 70°C) isolates from Octopus Spring also revealed different strategies for phosphorus assimilation: in contrast to OS-B′, OS-A lacks a C-P lyase operon and is unable to grow heterotrophically in the dark from phosphonates (26). Since focused studies of high-temperature *Synechococcus*-like A/B-lineage ecotypes have been largely limited to the thick laminated mats of Octopus Spring, Mushroom Spring, and Hunter’s Hot Springs, it is unknown whether they are driven by the same adaptations in other springs. Specifically, the degree to which pH influences temperature adaptation and taxonomic makeup of A/B-lineage cyanobacteria is understudied.

Here, we leveraged metagenomic sequencing of samples across a range of pH, temperature, geochemistry, and community morphology to further define the taxonomic distribution and genomic content of the A/B-lineage and other thermophilic cyanobacteria within YNP hot springs. We focused specifically on communities existing in a range of temperatures at circumneutral to alkaline pH and in communities with morphologies ranging from thin and thick mats to filaments. We asked whether the niches defined for the *Synechococcus*-like A/B lineage in Mushroom and Octopus springs are applicable to other Yellowstone hot springs and whether the distribution and relative abundances of phototrophic taxa are constrained by a combination of temperature and pH. Using a pangenome approach, we detail genomic comparisons of 15 *Synechococcus*-like A/B-lineage metagenome-assembled genomes (MAGs) from three distinct clades.

## RESULTS AND DISCUSSION

### Sample site descriptions and recovery of MAGs.

**FIG 1:**
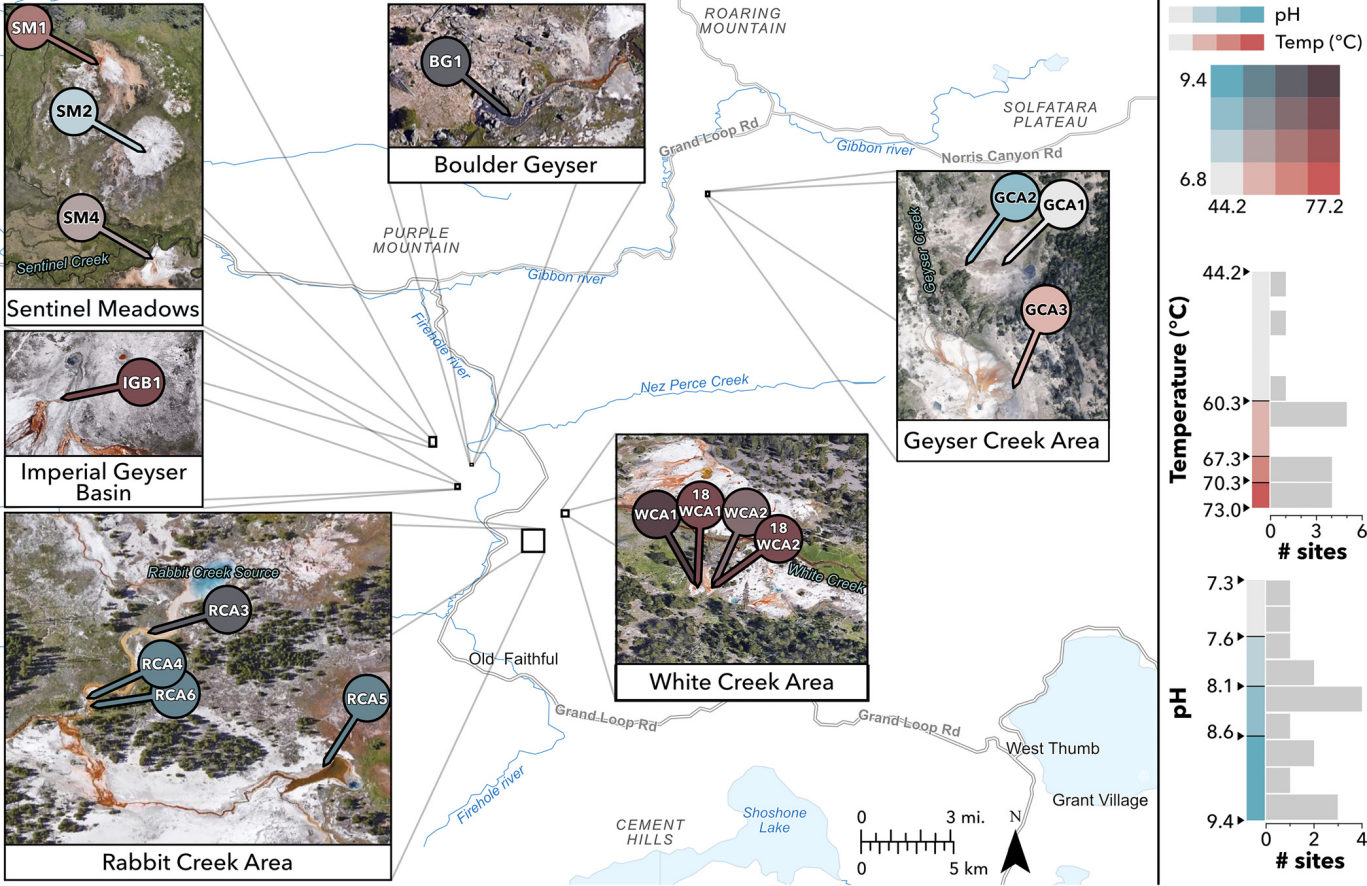
Sample locations. Samples were collected in Yellowstone National Park at locations with various temperatures, pH, and community morphology. Samples are shown sorted into 4 temperature (red) and 4 pH (blue) color coordinates. Sorting was done according to geometric intervals to account for skewed data and better differentiate temperatures near the upper limits. Research conducted under Yellowstone Research Permit YELL-2017-SCI-7020.

We asked whether the temperature constraints placed on laminated, cyanobacterial mats in alkaline Mushroom and Octopus springs were similar to those in other Yellowstone hot springs with varying geography, community morphology, and pH. Additionally, we examined the extent to which pH constrains the upper temperature limit and taxonomic composition of thermophilic cyanobacteria. We collected 16 samples from 11 hot springs within 3 geyser basins in 2017 and 2018 (Fig. 1; Table 1), targeting the transition between pigmented photosynthetic communities and chemotrophic zones, termed the photosynthetic “fringe.” YNP regions sampled include the Middle Geyser Basin (Rabbit Creek area [RCA]), Lower Geyser Basin (Boulder Geyser, Imperial Geyser Basin, Sentinel Meadows, White Creek area [WCA]), and Gibbon Geyser Basin (Geyser Creek area [GCA]). Generally, sample temperatures ranged from 44.2°C to 73.0°C, and pH ranged from 7.3 to 9.4. Sulfide concentrations ranged from 0.22 μM to 4.93 μM (with one exception at 18.99 μM), chloride from 7.7 μM to 12.0 μM, and iron from 93.8 nM to 765.3 nM. In samples taken from 2018, nitrate concentrations were detectable up to 14.3 μM, while ammonium concentrations were detected up to 6 μM. No fixed-nitrogen data were available for 2017 samples.

**TABLE 1:** Metagenome sample descriptions

Sample name^*a*^	Sample ID	IMG genome ID	BioSample accession	Total assembly length (bp)^*b*^	Gene count	pH	T (°C)	Community morphology
BG1	170629B	3300028893	SAMN26200800	196,918,189	436,827	8.7	68.5	Red mat and filaments
GCA1	170630B	3300038630	SAMN26200803	222,603,420	441,247	7.6	59.9	Orange/yellow mat
GCA2	170630C	3300040982	SAMN26200804	291,888,643	632,804	8.3	44.2	Pink/white/grey mat
GCA3	170630D	3300028820	SAMN26200805	213,192,026	395,775	7.3	62.7	Red/grey mat
IGB1	180603O	3300036711	SAMN26200807	117,128,738	264,866	8.2	66.6	Orange/yellow mat
RCA3	170626A	3300028606	SAMN26200794	114,093,415	252,304	9.1	68.4	Thin yellow/orange mat
RCA4	170626B	3300028609	SAMN26200795	195,353,357	412,629	9.2	62.3	Orange/yellow mat
RCA5	170626C	3300028611	SAMN26200796	108,943,318	208,402	9.4	62.4	Orange/yellow mat
RCA6	170626D	3300028818	SAMN26200797	182,682,112	386,452	9.3	62.3	Orange/yellow mat
SM1	170627A	3300028613	SAMN26200798	316,999,032	710,899	7.9	68.4	Thin yellow/orange mat
SM2	170627E	3300028839	SAMN26200799	475,303,863	1,088,432	8.1	51.4	Orange/yellow mat
SM4	180602L	3300038980	SAMN26200806	76,631,469	142,361	8.0	62.3	Orange/yellow mat
WCA1	170629F	3300028816	SAMN26200801	104,661,654	234,717	8.8	71.0	Green filaments
18WCA1	180603R	3300038981	SAMN26200808	101,090,071	215,532	8.4	73.0	Green filaments
WCA2	170629H	3300028617	SAMN26200802	131,707,792	292,784	8.6	69.4	Green filaments and mat
18WCA2	180603S	3300038484	SAMN26200809	142,901,704	282,903	8.2	71.9	Green filaments

a RCA, Rabbit Creek Area; SM, Sentinel Meadows; BG, Boulder Geyser; WCA, White Creek area; GCA, Geyser Creek area; IGB, Imperial Geyser Basin.

b A 150-bp minimum scaffold length was used.

In the Middle Geyser Basin, samples taken from the RCA consisted of yellow/orange mats. Samples RCA3, RCA4, and RCA6 were collected from Rabbit Creek, a fast-moving outflow in a length along the channel spanning ~70 to 150 m from the source spring and between 68.4 and 62.3°C. Sample RCA5 was collected at 62.4°C from a separate slow-moving outflow of Smoking Gun Spring, ~20 m from the source. pH values across all RCA samples were similar (9.1 to 9.4). RCA5 had the highest sulfide concentration (18.99 μM) compared to the other RCA samples, which ranged from 0.25 to 0.44 μM (Table 1; also, see Data Set S1 in the supplemental material).

10.1128/msystems.00317-22.6 DATA SET S1 Sample and metagenome assembly descriptions and metrics. Download Data Set S1, XLSX file, 0.02 MB. Copyright © 2022 Kees et al. 2022 Kees et al. https://creativecommons.org/licenses/by/4.0/ This content is distributed under the terms of the Creative Commons Attribution 4.0 International license.

In the Lower Geyser Basin, Sentinel Meadows (SM) samples were collected from shallow outflows of three separate springs, all close to their source (5 to 12 m downstream). Samples SM1, SM2, and SM4 all had pHs near 8, while temperatures were 68.4°C, 51.4°C, and 62.3°C, respectively (Table 1). WCA samples had temperatures among the highest (69.4 to 73°C) and consisted exclusively of communities with green filament structures (Table 1). Beyond community morphologies and temperatures, other distinguishing physical and geochemical parameters were not observed for WCA samples, although samples 18WCA1 and 18WCA2 were the only samples for which nitrate and ammonium concentration data are available. Nitrate was detectable in both samples (14.3 μM), while ammonium was below detection limit in 18WCA1 and 6.0 μM in 18WCA2 (Data Set S1). Micromolar concentrations of nitrate and/or ammonia have been previously reported in Lower Geyser Basin springs (31, 32), and thus we expect that 2017 samples would have had similar concentrations. It is possible that unmeasured parameters and/or biogeography are primary predictors of the characteristic extremely thermophilic green filaments in these springs. Imperial Geyser Basin samples include BG1 and IGB1. The BG1 sample, at pH 8.7 and 68.5°C, consisted of a red mat with filaments, while IGB1 was taken from an orange/yellow mat at pH 8.2 and 66.6°C. Samples in the Lower Geyser Basin had sulfide concentrations ranging from 0.22 to 4.93 μM.

In the Gibbon Geyser Basin, samples taken from the GCA consisted primarily of orange, pink, and red mats overlaid on black and gray sediments. Two samples (GCA1 and GCA2) were from a single shallow outflow within ~10 m of the source spring, while a third (GCA3) was from a shallow outflow further downstream of its source. Temperatures in the GCA ranged from 44.2 to 62.7°C and pH from 7.3 to 8.3 (Table 1). Sulfide concentrations ranged from 0.6 to 2 μM, while sulfate (1.2 to 1.4 mM) and chloride (11 to 13 mM) were elevated compared to those in other sample areas (~0.2 mM and 6 to 9 mM, respectively) (Data Set S1).

**FIG 2:**
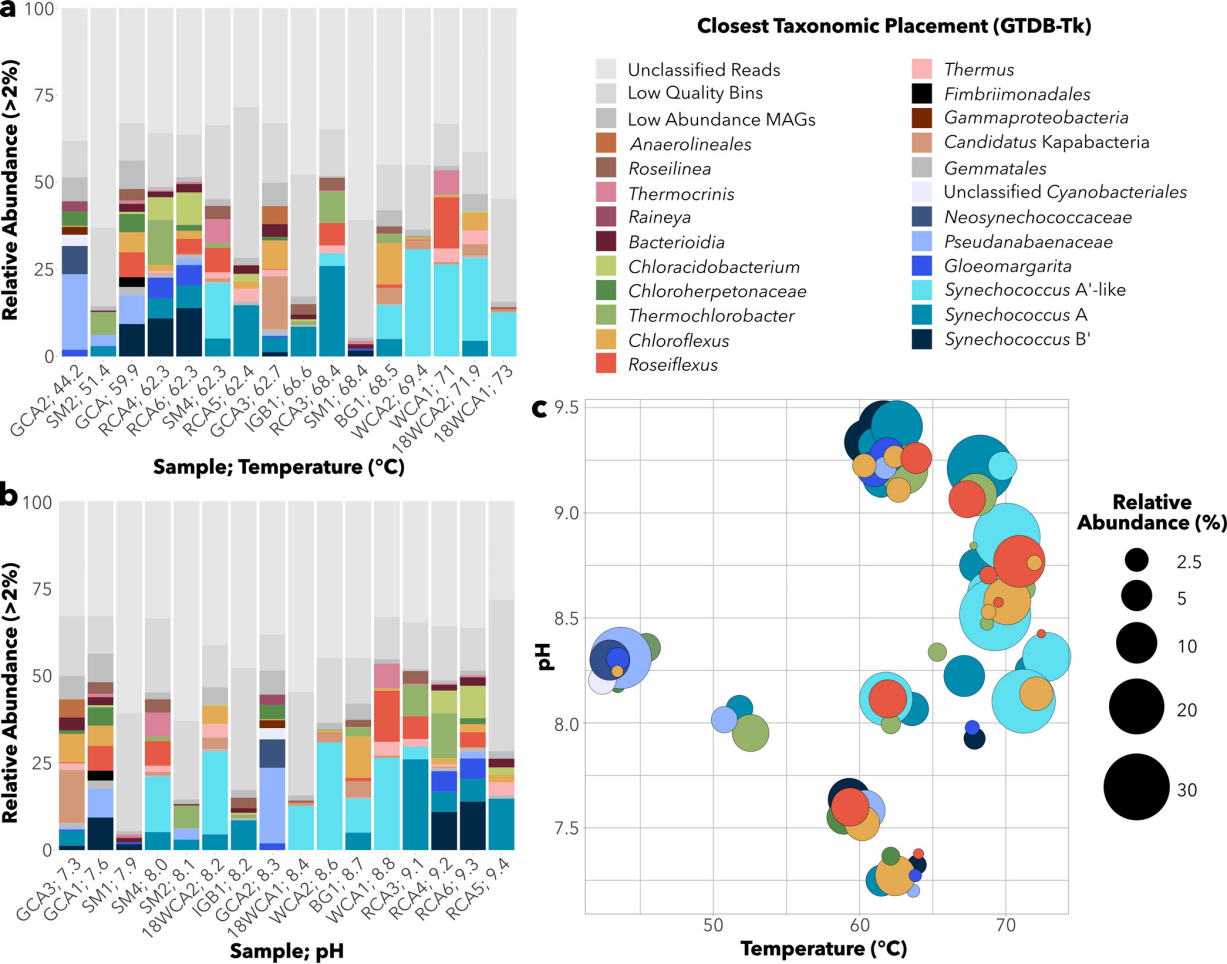
MAG abundance across samples. Relative abundance of all MAGs with >2% abundance in at least one sample. Samples are ordered by (a) temperature or (b) pH. (c) Abundances of phototrophic MAGs along combined pH and temperatures. Unmapped reads, low-quality bins, and low-abundance MAGs are grouped per sample (shown in gray).

A total of 360 metagenome bins were recovered in this study, 221 of which met minimum criteria to be considered medium- to high-quality MAGs (>50% completeness, <10% contamination) (as defined in reference 33). Taxonomic abundance estimates of MAGs revealed that *Cyanobacteria*, *Chloroflexi*, and *Chlorobi* were the most common photosynthetic phyla observed across samples (Fig. 2), consistent with previous work in YNP hot springs (14, 34). In total, 38 medium- to high-quality cyanobacterial MAGs were recovered (Table 2).

**TABLE 2:** Cyanobacterial MAG descriptions

MAG^*a*^	Relative abundance (%)	Completeness (%)	Contamination (%)	Taxonomic assignment	Closest reference genome^*b*^	ANI to reference (%)^*b*^	High quality^*c*^
RCA3.9B	3.51	64.47	0.52	*Synechococcus*-like A′ type	JA-3-3Ab (OS-A)	85.16	
BG1.9A	9.69	79.82	4.87	*Synechococcus*-like A′ type	JA-3-3Ab (OS-A)	87.29	Y
18WCA2.12A	23.65	81.58	4.39	*Synechococcus*-like A′ type	JA-3-3Ab (OS-A)	87.32	Y
WCA2.4	30.76	86.84	0	*Synechococcus*-like A′ type	JA-3-3Ab (OS-A)	87.3	Y
SM4.3A	15.86	94.74	0.88	*Synechococcus*-like A′ type	JA-3-3Ab (OS-A)	87.11	Y
WCA1.12	26.42	96.49	0	*Synechococcus*-like A′ type	JA-3-3Ab (OS-A)	87.47	Y
18WCA1.11	12.68	98.25	0	*Synechococcus*-like A′ type	JA-3-3Ab (OS-A)	87.47	Y
18WCA2.12B	4.48	51.27	0	*Synechococcus*-like A type	JA-3-3Ab (OS-A)	99.61	
RCA6.5B	6.51	55.78	0.44	*Synechococcus*-like A type	JA-3-3Ab (OS-A)	99.32	
BG1.9B	4.99	62.96	0	*Synechococcus*-like A type	JA-3-3Ab (OS-A)	99.24	
SM4.3B	5.14	64.04	0	*Synechococcus*-like A type	JA-3-3Ab (OS-A)	99.21	
RCA4.11B	5.81	72.37	0	*Synechococcus*-like A type	JA-3-3Ab (OS-A)	99.21	Y
SM2.9A	3.00	76.41	4.66	*Synechococcus*-like A type	JA-3-3Ab (OS-A)	99.31	Y
RCA3.9A	25.99	86.55	0.88	*Synechococcus*-like A type	JA-3-3Ab (OS-A)	99.15	Y
GCA3.42A	4.28	89.3	0.1	*Synechococcus*-like A type	JA-3-3Ab (OS-A)	99.23	Y
IGB1.12	8.54	91.23	0.1	*Synechococcus*-like A type	JA-3-3Ab (OS-A)	99.34	Y
RCA5.7	14.73	94.3	0.88	*Synechococcus*-like A type	JA-3-3Ab (OS-A)	99.16	Y
RCA6.5A	13.88	53.9	4.39	*Synechococcus*-like B′ type	NA	NA	
RCA4.11A	10.92	59.91	3.2	*Synechococcus*-like B′ type	JA-2-3B′(2-13) (OS-B′)	96.96	
SM1.7B	1.66	71.93	8.33	*Synechococcus*-like B′ type	JA-2-3B′(2-13) (OS-B′)	97.99	Y
GCA3.42B	1.22	72.19	0	*Synechococcus*-like B′ type	JA-2-3B′(2-13) (OS-B′)	98.12	Y
GCA1.24	9.32	91.23	1.75	*Synechococcus*-like B′ type	JA-2-3B′(2-13) (OS-B′)	97.93	Y
GCA2.22	1.13	98.72	0	A/B-lineage relative (family)	NA	NA	Y
SM2.3A	0.57	82.32	7.42	A/B-lineage relative (order)	NA	NA	Y
RCA6.18	5.85	62.73	3.42	*Gloeomargarita* sp.	*G. lithophora* Achichica-D10	79.93	
GCA2.30	1.94	64.14	0	*Gloeomargarita* sp.	*G. lithophora* Achichica-D10	83.51	
RCA4.1	5.88	64.67	1.71	*Gloeomargarita* sp.	*G. lithophora* Achichica-D10	79.91	
GCA3.22	0.39	84.62	0	*Gloeomargarita* sp.	*G. lithophora* Achichica-D10	79.43	Y
SM1.2	0.56	86.05	2.32	*Gloeomargarita* sp.	*G. lithophora* Achichica-D10	79.2	Y
GCA1.18	0.53	74.21	0.59	*Pseudanabaenaceae* bacterium	*Pseudanabaenaceae* M5B4	77.84	Y
GCA2.3	21.65	88.09	0.24	*Pseudanabaenaceae* bacterium	*Pseudanabaenaceae* M5B4	78.63	Y
SM2.6A	0.68	89.27	1.65	*Pseudanabaenaceae* bacterium	NA	NA	Y
SM2.6B	2.48	74.4	5.07	*Pseudanabaenaceae* bacterium	NA	NA	Y
RCA6.13	1.89	91.04	0.47	*Pseudanabaenaceae* bacterium	NA	NA	Y
GCA3.6	0.43	95.28	0.63	*Pseudanabaenaceae* bacterium	NA	NA	Y
GCA1.15	7.77	95.4	2.67	*Pseudanabaenaceae* bacterium	NA	NA	Y
GCA2.36	3.22	94.52	0.67	*Cyanobacteriales* bacterium	*Cyanobacteriales* bacterium JAAUUE01	75.88	Y
GCA2.6	8.12	85.26	0.24	*Neosynechococcaceae* bacterium	*Neosynechococcaceae* GCF-001939115	75.91	Y

a RCA, Rabbit Creek Area; SM, Sentinel Meadows; BG, Boulder Geyser; WCA, White Creek area; GCA, Geyser Creek area; IGB, Imperial Geyser Basin.

b NA, data not available.

c Y, yes.

### Recovery of novel *Synechococcus*-like A/B-lineage MAGs from the upper temperature limit of photosynthesis.

Our primary focus in this study was *Synechococcus* A/B-lineage (genus-level) MAGs, as they were present across nearly all sites (Fig. 2). A/B-lineage MAGs were recovered from all samples between 51.4°C and 73°C, with three distinct taxa identified by the Genome Taxonomy Database Toolkit (GTDB-Tk) (35). Two other MAGs classified at the order and family level with the A/B lineage were recovered in low relative abundance from the two lowest-temperature samples (GCA2.22 = 1.13% at 44.2°C; SM2.3A = 0.57% at 51.4°C).

**FIG 3:**
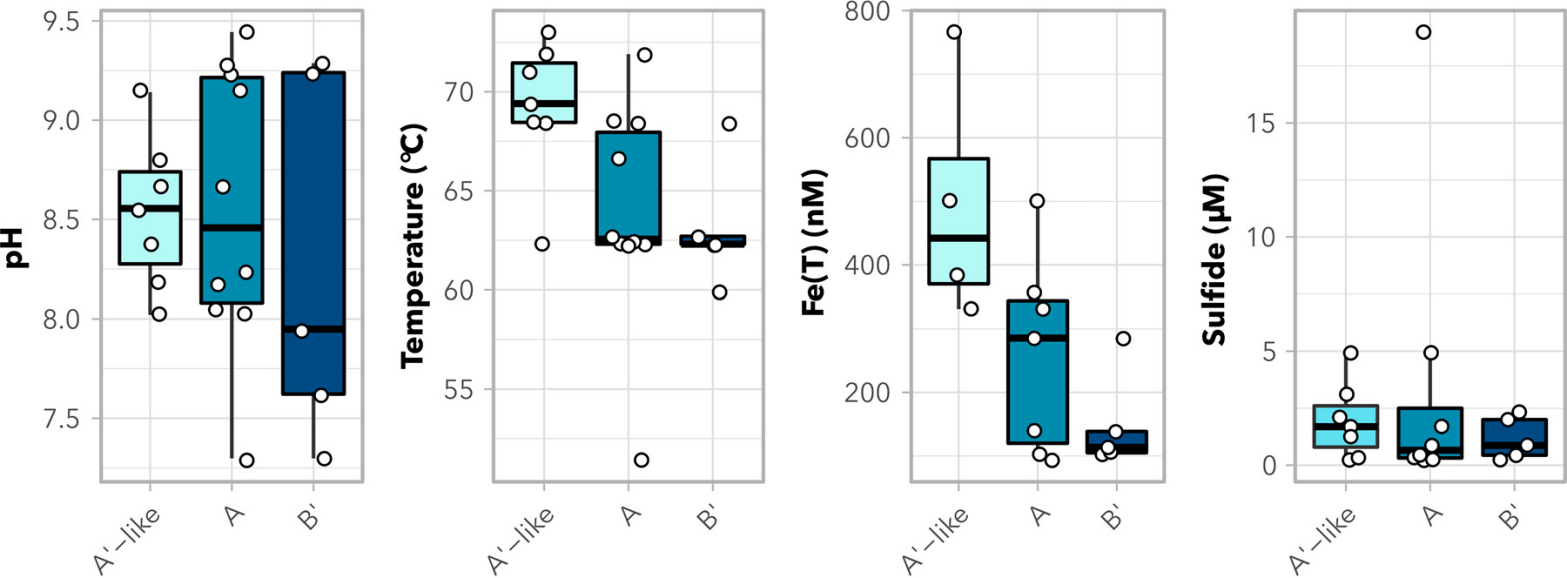
*Synechococcus*-like A/B-lineage MAG ranges with environmental parameters. Presence of cyanobacterial MAGs by taxon across pH, temperature, total iron, and sulfide concentrations in sample water. Box-and-whisker plots represent medians, 25 to 75% interquartile ranges, and ranges of values. Dots represent parameters associated with individual MAGs.

Of 22 total medium- to high-quality A/B-lineage MAGs (Table 2), 10 had greater than 99% average nucleotide identity (ANI) with the closest reference genome from strain OS-A. Meanwhile, five MAGs had ~96 to 98% ANI with OS-B′, suggesting relatively higher diversification among B′ MAGs recovered (Data Set S2). MAGs assigned to the A taxon were recovered from temperatures between 51.4 and 71.9°C, while B′ MAGs occupied temperatures between 59.9°C and 68.4°C but were most abundant below 62.3°C (Fig. 2a and 3). The remaining seven A/B-lineage MAGs recovered from 62.3 to 73.0°C samples had ~85 to 88% ANI to the OS-A reference genome. MAGs representing this taxon were most abundant at the highest temperature range in our samples (69 to 73°C; Fig. 2a), suggesting they are A′-like ecotypes (15, 16). Two MAGs (SM2.3A and GCA.22) were classified near the A/B-lineage at the order or family level but had no closely related reference genome available. A phylogenomic analysis using 120 concatenated marker genes (the GTDB bac120 set) (36) from 38 cyanobacterial MAGs and 79 reference genomes showed that SM2.3A and GCA2.22 each branched individually from the A/B lineage while also confirming clustering of the three A/B-lineage clades (Fig. S1).

10.1128/msystems.00317-22.1 FIG S1 Phylogenomic tree of cyanobacterial MAGs. Distances were calculated from an aligned, concatenated set of 71 conserved marker genes from 26 high-quality (>70% completeness and <10% contamination) cyanobacterial MAGs and a set of reference genomes. Node symbols denote bootstrap values above 80 from 100 bootstraps within RAxML. Download FIG S1, TIF file, 1.9 MB. Copyright © 2022 Kees et al. 2022 Kees et al. https://creativecommons.org/licenses/by/4.0/ This content is distributed under the terms of the Creative Commons Attribution 4.0 International license.

10.1128/msystems.00317-22.7 DATA SET S2 MAG descriptions and metrics. Download Data Set S2, XLSX file, 0.1 MB. Copyright © 2022 Kees et al. 2022 Kees et al. https://creativecommons.org/licenses/by/4.0/ This content is distributed under the terms of the Creative Commons Attribution 4.0 International license.

To better define the taxonomy of the suspected A′-like clade of MAGs, we performed a phylogenetic analysis of 11 PsaA and 16 RbcL protein sequences recovered from A/B-lineage MAGs. PsaA sequences recovered from our putative A′-like MAGs cluster with sequence PEA9 from another putative A′-like ecotype in YNP (24) (Fig. S2). RbcL sequences recovered from A′-like MAGs cluster with a *Synechococcus*-like isolate, strain OH28, which has also been classified as an A′-like ecotype (30). These observations suggest that this highest-temperature clade of *Synechococcus*-like A/B-lineage MAGs most likely constitutes a A′-like ecotype, for which no genomes had been previously sequenced.

10.1128/msystems.00317-22.2 FIG S2 Support for taxonomic assignment of A′-like MAGs. (a) PsaA amino acid sequences extracted from 11 A/B-lineage MAGs compared with a subset of those published in reference 10. (b) 16 RbcL amino acid sequences extracted from 13 A/B-lineage MAGs compared to a subset published in reference 30. Node symbols denote bootstrap values above 80 from 100 bootstraps within RAxML. Each sequence is named and colored according to MAG and taxonomic assignment. OS-A, OS-B′, and other published PsaA and RbcL sequences are in black. Download FIG S2, TIF file, 1.1 MB. Copyright © 2022 Kees et al. 2022 Kees et al. https://creativecommons.org/licenses/by/4.0/ This content is distributed under the terms of the Creative Commons Attribution 4.0 International license.

### Recovery of *Pseudanabaenales* MAGs and novel thermophilic *Gloeomargarita* MAGs.

Two other sets of cyanobacterial MAGs that we recovered belong to *Pseudanabaenaceae* and *Gloeomargarita* taxa. A total of seven *Pseudanabaenaceae* MAGs were recovered from samples up to 62.7°C and pH 7.3 to 9.3 in the Geyser Creek area, Rabbit Creek area, and Sentinel Meadows. Two MAGs placed within the family *Pseudanabaenaceae*—GCA1.18 and GCA2.3—had ~78% ANI to cyanobacterial MAG M5B4 recovered from a hot spring in Lakash, India (37) (Fig. S1; Table 2). Five other MAGs—GCA3.6, GCA1.15, SM2.6A, SM2.6B, and RCA6.13—had unknown taxonomic placement within *Pseudanabaenaceae*. Phylogenomic analysis placed GCA3.6, GCA1.15, SM2.6A, and RCA6.13 in a single clade (Fig. S1); none were closely associated with any of the seven included *Pseudanabaenaceae* reference GTDB genomes.

A total of five MAGs were assigned to the genus *Gloeomargarita*, with ~79 to 82% ANI to the closest reference genome, Gloeomargarita lithophora Alchichica-D10 (38, 39). Phylogenomic analysis placed *Gloeomargarita* MAGs in two clades associated with Alchichica-D10 (Fig. S1). Four MAGs formed a cluster, while the fifth MAG (GCA2.30) was alone but more closely associated with Alchichica-D10. *Gloeomargarita* MAGs were recovered from samples with temperatures up to 68.4°C and from samples between pH 7.3 and 9.3 (Fig. 2) but were most abundant (>2%) at pH 9.2 to 9.3. This distribution is consistent with previous 16S rRNA amplicon results indicating the presence of *Gloeomargarita* operational taxonomic units (OTUs) up to 70.8°C in Rabbit Creek and Bison Pool (11). In comparison, the *G. lithophora* Alchichica-D10 isolate has a growth range of 15 to 30°C in the lab (38, 39). Considering *Gloeomargarita* is an early branching genus of cyanobacteria (38–40), recovery of MAGs (and potentially future isolates) at such high temperatures, may yield new insights into the evolution of thermophily in cyanobacteria.

### Influence of environmental parameters on A′-like MAG distributions.

Temperature, pH, and sulfide have all been demonstrated to be the primary environmental constraints on photosynthesis in hot springs (8, 41, 42). However, in our samples, the only environmental parameters that impacted the distribution of medium- to high-quality A′-like versus B′-like MAGs were temperature (adjusted *P* = 0.0249; Dunn’s test with Bonferroni correction) and total Fe concentration (adjusted *P* = 0.0149) (Fig. 3). Sulfide concentration did not predict the distribution of A′-like MAGs compared to other A/B-lineage MAGs (adjusted *P* = 0.8469). A′-like MAGs were generally recovered from outflows with higher temperatures (62 to 73°C) than B′ MAGs (60 to 68°C). Differences in temperature ranges between A′-like and B′ MAGs are consistent with previous work showing shifted, yet overlapping, temperature ranges among B′, A, and A′ ecotypes, both *in situ* and in laboratory cultures (15, 17, 27). A′-like MAGs were also recovered from samples with higher total iron [Fe(T)] concentrations [383 to 501 nM Fe(T)] than B′ MAGs [101 to 285 nM Fe(T)]. It is possible that the impacts of temperature and iron on the distribution of A′-like MAGs are interlinked. Since iron is essential for the construction of photosystem II (PS II) and chlorophyll synthesis and is a cofactor for PS I and the photosynthetic electron transport chain, cyanobacteria require an estimated 10-fold-higher intracellular concentration than similarly sized nonphotosynthetic bacteria (43–45). A higher total iron concentration in samples where A′-like MAGs were recovered than in other samples in the study might suggest higher demands for PS repair and turnover in cyanobacteria near the upper temperature limit, potentially due to increased heat stress on the photosynthetic apparatus.

Beyond differences in iron concentration and temperature, A′-like MAGs were recovered primarily from filament communities. These include four green filament samples (WCA1, WCA2, 18WCA1, and 18WCA2) and one red mat/filament sample (BG1). A′-like MAGs were also recovered from two of the orange mats (RCA3 and SM4) (Tables 1 and 2; Data Set S1). B′ MAGs were recovered only from mat communities, while A MAGs were recovered from eight mat communities and two filament communities. Given that green filament samples containing A′-like MAGs were observed only in White Creek Area samples, we expected biogeography to impact phylogenomic distance among A′-like, A, and B′ clades. An influence of biogeography on variation in *psaA* genes among A/B-lineage organisms was demonstrated previously (10). In our data set, geographic distance was not significantly predictive of phylogenomic distance among A/B-lineage MAGs (Mantel test; *P* = 0.095, *r* = 0.135). This result suggests that dispersal barriers were not a factor in the distribution of A/B-lineage MAGs across YNP. Thus, temperature and iron concentration likely play primary roles in the distribution of A′-like MAGs.

A′-like MAGs were recovered only from pH of 8.0 to 9.1, in contrast to A MAGs (7.3 to 9.4) and B′ MAGs (7.3 to 9.3) (Fig. 2b and Fig. 3), suggesting that one or multiple physiological components have narrower pH constraints in A′-like MAGs, perhaps as a consequence of adaptation to the highest temperatures at which phototrophy has been observed. In general, while sample size is limited, all phototrophic fringe communities observed above 70°C in this study were limited to pH 8.1 to 8.8 (Fig. 2c). Similar constraints on phototrophs observed above 70°C were previously seen across much larger sample sets within YNP (8, 14). In those studies, *chlL*/*bchL* and *bchY* amplicons at sample temperatures between 65°C and 70°C were observed between pH 5.5 and pH 9.5, while amplicons above 70°C were more limited to pH ~7.5 to 9.2.

### Pangenomes of A/B-lineage metagenome-assembled and reference genomes.

Given that A/B-lineage taxa were the most widely distributed among cyanobacterial MAGs we recovered and that each phylogenomically distinct clade occupies a different temperature range, we narrowed our focus to A/B-like MAGs to elucidate shared and unique genome content among them. The pangenome analyses were conducted on 15 A/B-lineage MAGs with >70% completeness and <10% contamination (based on assessment of MAG quality by CheckM [46]). We removed the MAGs below these thresholds to decrease the likelihood of including MAGs with spuriously missing genes due to metagenome assembly and binning errors. These criteria have been used in genome-wide comparisons of MAGs elsewhere (47).

**FIG 4:**
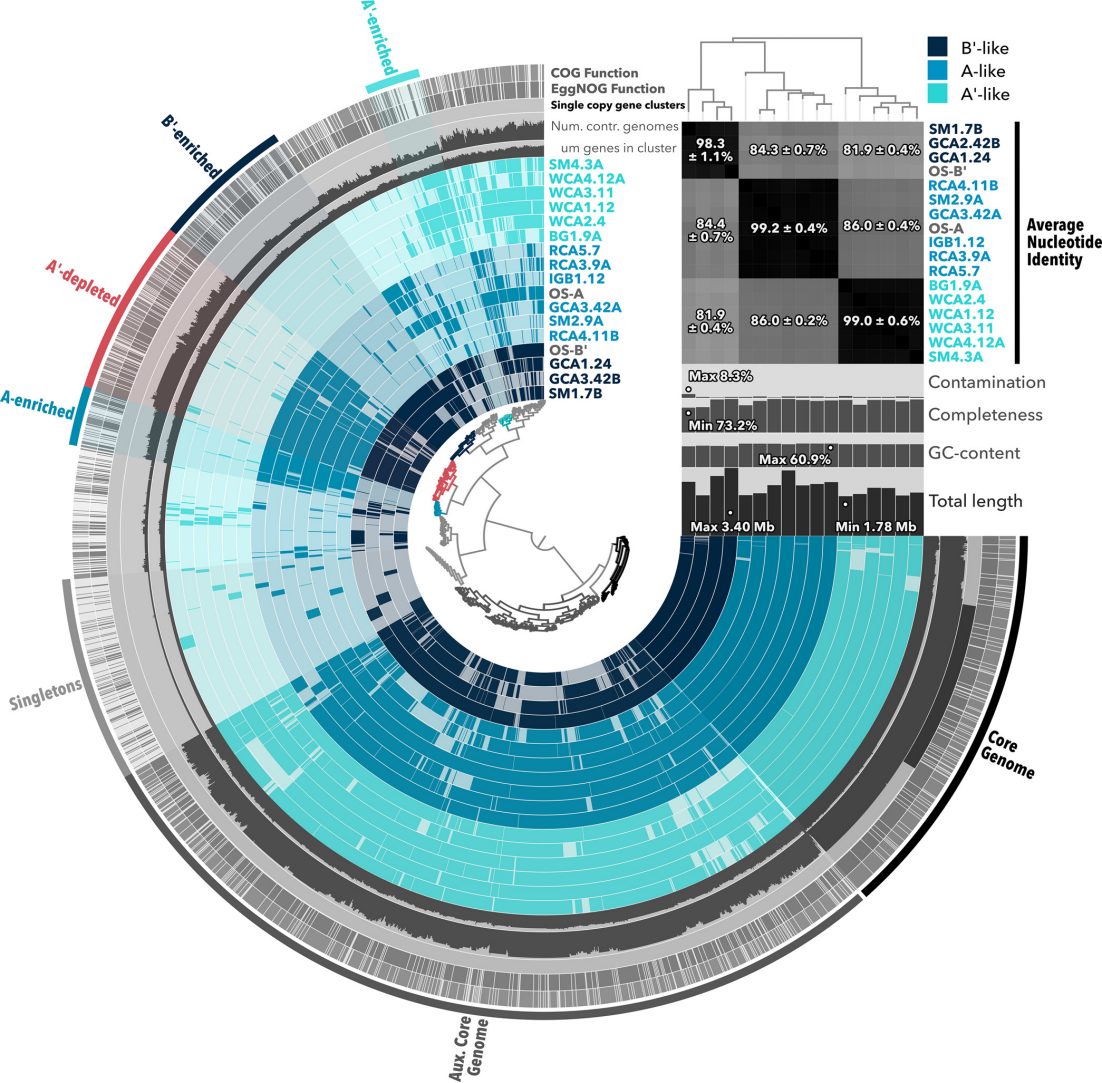
Pangenome of *Synechococcus*-like A/B-lineage MAGs. Gene clusters present in 15 A/B-lineage MAGs and two reference genomes are shown in colored rings. The central dendrogram depicts ordering of gene clusters by frequency and was used to select gene clusters unique to or enriched in different MAGs or clades (outermost rings and highlighted gene cluster selections). Genomes were ordered based on a phylogenomic tree produced in RAxML. Percent average nucleotide identity comparisons are statistically represented on a clade-by-clade basis. Contamination and completion were calculated by checkM and the anvi’o pangenomics suite, and ANI was calculated by pyANI within anvi’o. NCBI COGs were identified using the 2020 release within anvi’o, and EggNOG functions were predicted based on the 5.0.1 database.

We performed pangenome analysis within anvi’o (48) using A/B-lineage MAGs and the OS-A and OS-B′ reference genomes. The pangenome (Fig. 4) was composed of a total of 3,274 gene clusters, with a core genome of 489 universally shared clusters. An auxiliary core of 1,229 gene clusters was shared by multiple representatives in each of the A′-like, A, and B′ clades. Of particular interest were gene clusters that were unique to or absent from A′-like MAGs, given that they are capable of occupying the thermal limit of photosynthesis and that they form a separate clade from A and B′ genomes (Fig. S1).

### Recovered A′-like MAGs do not encode nitrogenase or protochlorophyllide reductase (DPOR).

**FIG 5:**
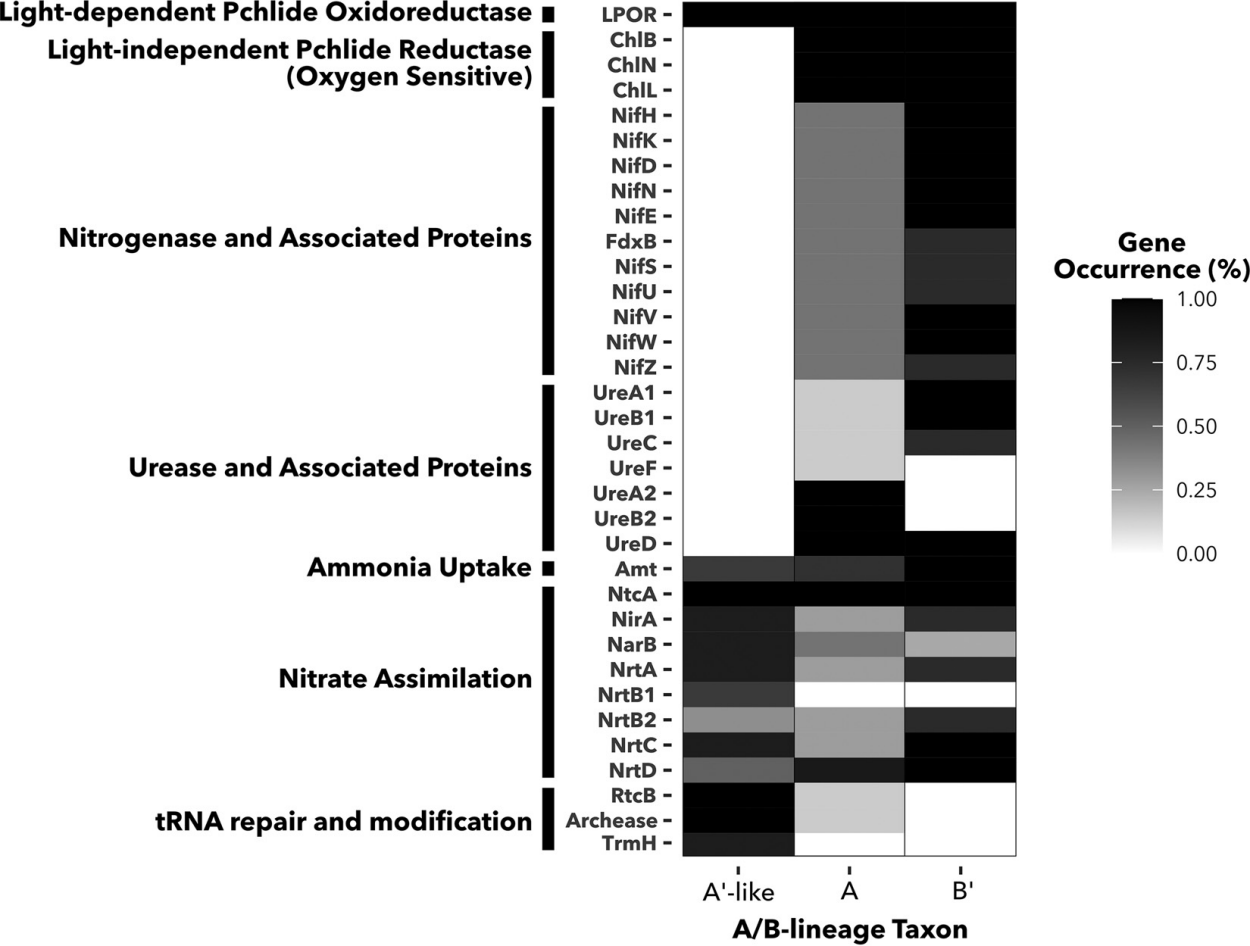
Occurrence of chlorophyllide synthesis, nitrogen metabolism, and tRNA repair and modification genes in A/B-lineage taxa. Heat map fill indicates percentage of A′-like (*n* = 6), A (*n* = 7), and B′ (*n* = 4) MAGs in which each gene cluster is present. Gene clusters are labeled by names of predicted proteins they encode and ordered by function.

To examine potential gene gain or loss within A′-like strains that may suggest adaptation to higher temperatures, a functional enrichment analysis was performed within anvi’o (49). Here, the term “enriched” is used when a gene cluster or a set of gene clusters is present more often in one clade of MAGs (A′-like, A, or B′) than the others and supported by an adjusted *q* value of <0.05 in the functional enrichment analysis. Likewise, a gene cluster is considered depleted in a clade when it is found more often in two others. More gene clusters were depleted (258 clusters) than enriched (80 clusters) in the A′-like clade, which suggests a streamlining of the A′-like genome to accommodate a high temperature range. These results are consistent with a general trend previously noted for thermophilic cyanobacteria, including the A/B lineage (50, 51). Gene clusters depleted in A′-like MAGs include those predicted to encode nitrogenase—*nifH*, *nifD*, and *nifK* (Data Set S3). Although functional enrichment scores for individual *nif* genes were just above the 0.05 threshold to be considered statistically significant (*q* = 0.0514), a consistent qualitative pattern emerges in the representation of *nif* operons and accessory genes across MAGs (Fig. 5; Table S1). OS-A and OS-B′ reference genomes and at least two MAGs assigned to each of these clades encode a nitrogenase enzyme, while these genes were absent from the A′-like clade. Other genes necessary for nitrogenase assembly are similarly absent from A′-like MAGs.

10.1128/msystems.00317-22.4 TABLE S1 Nitrogen and protochlorophyllide reductase proteins encoded in the A/B-lineage pangenome (15 MAGs and 2 reference genomes). Download Table S1, DOCX file, 0.02 MB. Copyright © 2022 Kees et al. 2022 Kees et al. https://creativecommons.org/licenses/by/4.0/ This content is distributed under the terms of the Creative Commons Attribution 4.0 International license.

10.1128/msystems.00317-22.8 DATA SET S3 A/B-lineage pangenome gene cluster enrichment data. Download Data Set S3, XLSX file, 0.3 MB. Copyright © 2022 Kees et al. 2022 Kees et al. https://creativecommons.org/licenses/by/4.0/ This content is distributed under the terms of the Creative Commons Attribution 4.0 International license.

These results suggest that A′-like cyanobacteria adapted to the upper temperature limits of photosynthesis must rely on fixed nitrogen, which can come from communities as well as the overflowing water. Unlike A and B′ MAGs and reference genomes, which encode clade-specific variants of urease (25), no urease genes were recovered from A′-like MAGs (Fig. 5). The ammonium transporter Amt along with a full suite of nitrate assimilation genes were present in the A′-like clade, suggesting that nitrate and/or ammonia could be the primary nitrogen sources for these organisms. Genes encoding nitrogenase were present in all metagenomes where A′-like MAGs were recovered except the highest-temperature sample (18WCA1, 73°C). In 18WCA1, the only nitrogenase genes present in the metagenome assembly were a *nifHBDK* cassette from *Roseiflexus*. No associated maturation genes were present, consistent with the genomes of *Roseiflexus* isolates, for which no evidence of diazotrophy has been observed (52, 53). However, micromolar nitrate detected in 18WCA1 suggests that the overflowing water may provide bioavailable nitrogen. Thus, we expect the overflowing water and/or diazotrophy by other members of the community to be the most likely sources of fixed nitrogen for A′-like MAGs.

The genes *chlLNB*, which encode the dark-operative DPOR, an oxygen-sensitive enzyme sharing evolutionary history with nitrogenase, were also absent in A′-like MAGs. Conversely, *chlLNB* were present in all A-type and B′-type MAGs and in the OS-A and OS-B′ reference genomes. In the absence of DPOR, the oxygen-tolerant light-dependent protochlorophyllide oxidoreductase (LPOR) is essential for chlorophyllide synthesis. Accordingly, the gene encoding LPOR, *por*, was found in all A/B-lineage MAGs and reference genomes, including A′-like MAGs. We can only speculate on the mechanisms that drove the A′-like clade to lose both nitrogenase and DPOR. Given the oxygen-sensitive nature of nitrogenase and DPOR, a loss of both enzymes in A′-like MAGs might suggest a loss of the ability to protect them. However, given the short distance of these samples from the source spring, low solubility of oxygen at these high temperatures (68 to 73°C), and ability of both enzymes to function at night when mats are anoxic (54, 55), we do not expect oxygen sensitivity to be an evolutionary force driving their loss. Instead, since nitrate/ammonium is available in the overflowing water in these sites and nitrogenase/DPOR-encoding genes are present in the metagenomes, we expect loss of nitrogenase and DPOR to be most likely due to their dispensability. The evolutionary history of diazotrophy is replete with examples of intra- and interphylum gene transfers as well as trait loss (56–58). Similar trait loss of DPOR is common to many photosynthetic taxa, often associated with maintenance of multiple *por* genes (59, 60). Loss of these genes in the highest temperature clade of A/B-lineage MAGs is also consistent with a general trend toward genome reduction in thermophiles (50, 51).

### The A′-like clade of MAGs is enriched for noncyanobacterial tRNA damage response proteins.

Two gene clusters enriched in the A′-like clade of the A/B-lineage pangenome are those encoding a tRNA-splicing ligase, RtcB, a predicted archease involved in RtcB activation, and a tRNA methyltransferase, TrmH (Data Set S3). Archease and RtcB are conserved across all domains of life (61) and are thought to assist in recovery from stress-induced tRNA damage (62, 63), while TrmH is implicated in regulating transcriptional response to stress conditions in Escherichia coli (64). Archease sequences were recovered only from A′-like MAGs and the OS-A genome. A BLASTp search of A′ RtcB against the NCBI nr database returned hits belonging to a small group of A/B-lineage genomes (~96% identity over 100% query coverage), including the OS-A genome, followed by RtcB sequences belonging to members of the family *Methylothermaceae* (~75 to 76% identity over 100% query coverage). BLASTp searches of A′-like archease against the full nr database produced similar results. These results suggest that RtcB and archease in the A/B lineage are highly diverged from those in other cyanobacteria. The top hit resulting from a BLASTp search for A′-like RtcB against all other cyanobacteria was for a tRNA-splicing ligase in a *Cyanobium* species at ~60% identity over 100% query coverage, while other hits were below 50% identity. Furthermore, the top hit for A′-like archease against cyanobacteria outside the A/B-lineage had only ~30% identity over 99% gene coverage. These results indicate that archease and RtcB in the A/B-lineage diverged from other cyanobacteria early in their history. Similarly, top hits from A′-like TrmH sequences searched against the NCBI nr database were from *Firmicutes*, *Proteobacteria*, *Actinobacteria*, *Chloroflexi*, and the *Fibrobacteres*/*Chlorobi*/*Bacteriodetes* (FBC) superphylum. Meanwhile, no significant similarity was found in BLASTp searches of A′-like TrmH against strains OS-A and OS-B′. Altogether, these results suggest a difference in tRNA repair and modification in response to stress between A′-like organisms and other A/B lineages, perhaps acquired through horizontal gene transfer.

### Functional divergence of conserved genes in *Synechococcus*-like MAGs.

**FIG 6:**
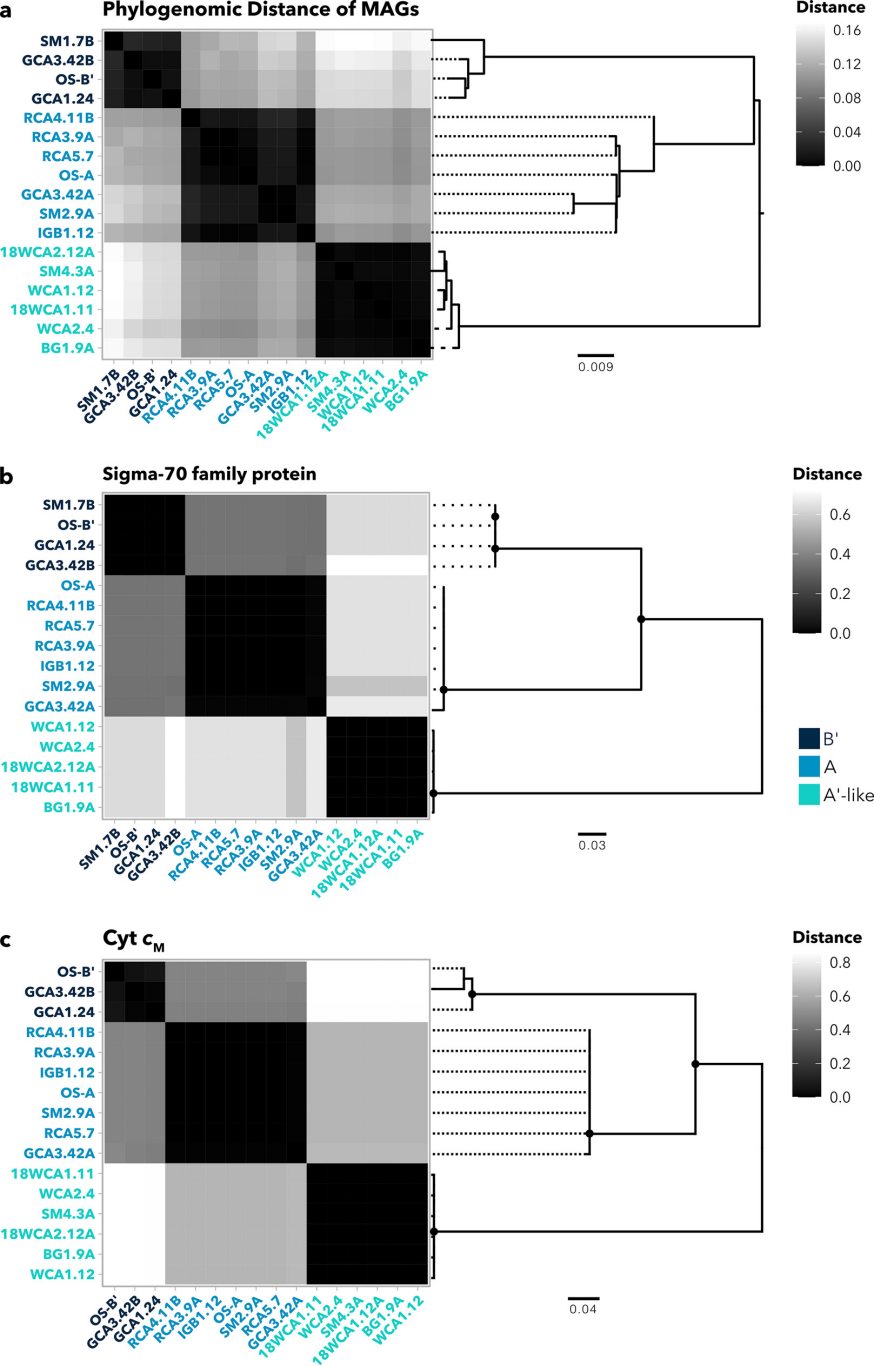
Phylogenetic distances of select gene clusters. (a) Phylogenomic distance heat map and corresponding tree using an aligned, concatenated set of 71 conserved marker genes from the A/B-lineage pangenome. (b) Phylogenetic distances of *cytM* genes extracted from A/B-lineage MAGs. (c) Uncharacterized sigma-70 family protein extracted from A/B-lineage MAGs and reference genomes. Node symbols denote bootstrap values above 80 from 100 bootstraps within RAxML. Each MAG is colored according to its taxon (A′-like, A, and B′).

We asked whether any genes present in A′-like MAGs diverged from genomes in the A and B′ clades in ways that might indicate adaptation to higher temperatures. Among gene clusters that were conserved across clades, we queried those without paralogs, with functional homogeneity (defined by point mutations) below 85% and geometric homogeneity (defined by gaps) above 85%. We calculated phylogenetic distance matrices of the 22 annotated gene clusters that met these criteria and compared them to distance matrices produced from a phylogenomic tree of 71 conserved marker genes in A/B-lineage MAGs (Fig. 6a; Table S2). We then sought gene clusters with a pattern of divergence that places A′-like sequences further away from A and B′ sequences than would be predicted by the phylogenomic tree, a pattern that would be suggestive of functional adaptation in the A′-like clade. One such cluster encodes a transcription factor in the sigma-70 family, which displays a clear pattern of divergence of A′-like sequences away from A and B′ sequences (Fig. 6b). Although this protein is uncharacterized, its divergence from other A/B-lineage types represents a potential regulon that is specialized in A′-like organisms.

10.1128/msystems.00317-22.5 TABLE S2 Proteins encoded by 71 single-copy bacterial marker genes used for phylogenomic analysis. Download Table S2, DOCX file, 0.01 MB. Copyright © 2022 Kees et al. 2022 Kees et al. https://creativecommons.org/licenses/by/4.0/ This content is distributed under the terms of the Creative Commons Attribution 4.0 International license.

Another gene cluster with high phylogenetic distance between the A′-like clade and the other two A/B-lineage clades encodes cytochrome *c*_M_ (Cyt *c*_M_) (Fig. 6c), which is present across cyanobacterial taxa but whose physiological role is still debated (65, 66). Transcription of *cytM* increases under conditions such as low temperature, high-intensity light, oxidative stress, osmotic stress, and nitrogen and sulfur limitation (67, 68). It has been suggested that under these stress conditions, Cyt *c*_M_ either plays a regulatory role or alleviates photosynthetic overreduction of electron carriers (65, 69, 70). Furthermore, studies in *Synechocystis* 6803 have shown that Cyt *c*_M_ suppresses both heterotrophy in the dark and photosynthesis during photomixotrophy (66, 70), supporting the notion that it performs a regulatory function. Divergence in Cyt *c*_M_ between A′-like MAGs and other A/B-lineage genomes may suggest that they differ in their response to stress conditions. Since A′-like populations often occupy environments at the absolute limits of photosynthesis, it is possible that temperature fluctuations require faster or stronger suppression of photosynthesis. Alternatively, since *cytM* expression is induced under N limitation, it is possible that a reliance on bioavailable nitrogen from overlying water or neighboring microbes may have required modification of the Cyt *c*_M_ regulon. Similar to phylogenomic distances, a clear influence of biogeography on intraclade variation of sigma-70 factor or Cyt *c*_M_ was not observed, particularly among sequences from the A′-like clade. Since intraclade variation was small, we conclude that variation observed is clade specific. Considering their potential to affect responses to environmental fluctuations, divergence in both Cyt *c*_M_ and this sigma-70 factor may guide future transcriptomic work into differences in stress responses between A/B-lineage clades in YNP hot springs. For a full summary of gene clusters, including annotations, functional/geometric homogeneity scores, and amino acid sequences, see Data Set S4.

10.1128/msystems.00317-22.9 DATA SET S4 A/B-lineage pangenome gene cluster descriptions and statistics. Download Data Set S4, XLSX file, 3.7 MB. Copyright © 2022 Kees et al. 2022 Kees et al. https://creativecommons.org/licenses/by/4.0/ This content is distributed under the terms of the Creative Commons Attribution 4.0 International license.

### Conclusions.

Cyanobacteria in Yellowstone hot springs have been widely studied for decades, yet much of the work has focused on thick laminated mats in only two hot springs within the same geyser basin. Here, we expanded this large body of work to include metagenome assembled genomes recovered from hot springs spanning different regions of YNP, with various temperatures, pH, geochemistry, and community morphologies. Particularly, our work focused on genomic divergence among three clades (A, B′, and A′-like) of *Synechococcus*-like A/B-lineage MAGs, adding to existing literature on differentiation within this lineage. Using phylogenetic trees constructed from aligned PsaA and RbcL sequences, we found that six A/B-lineage MAGs were most closely related to A′-like ecotypes previously found in YNP and Hunter’s Hot Springs. These A′-like MAGs, which were recovered from the highest temperatures in our samples, have not been successfully cultured or fully sequenced to date, and recovery of multiple MAGs provides new potential to uncover adaptations to the thermal limits of photosynthesis. We found that within the samples tested, both temperature and total Fe controlled the range of A/B-lineage MAGs, while the pH range of A′-like MAGs was narrow in comparison to the two other clades, suggesting that pH limits adaptations to high temperatures within these organisms. We uncovered genes that were enriched or depleted in each clade of A/B-lineage MAGs. Specifically, genes encoding both the light-independent protochlorophyllide reductase (DPOR) and nitrogenase were entirely absent from A′-like MAGs, suggesting that A′-like organisms must rely on fixed nitrogen from their environment, either from the overflowing water or from other diazotrophs in the community. Finally, we uncovered genes that diverge between clades of the A/B lineage, particularly Cyt *c*_M_, which provides an avenue for future work into the regulation of photosynthesis under thermal stress and nutrient-limited conditions in these organisms. Both the genome content of A′-like MAGs and environmental context will underpin future efforts to isolate this elusive clade. Overall, our work serves as an entry point for future hypothesis-based approaches to uncover physiological distinctions between YNP hot spring cyanobacterial taxa.

## MATERIALS AND METHODS

### Sample collection, sequencing, metagenome assembly, and binning.

Eleven sites throughout Yellowstone National Park were sampled, with 16 total samples collected for metagenomic analysis. Sample temperatures ranged from 44°C to 73°C, and pH ranged from 7.3 to 9.4. Generally, the border between pigmented mats or filaments and the unpigmented chemotrophic zone was targeted for sequencing, with a variety of microbial structures sampled, thin and relatively thick mats to filaments. Samples for DNA extraction and water analysis were collected as previously described (14). Briefly, pH and temperature were measured using a WTW pH 3310 meter (Xylem Analytics, Weilheim, Germany). A YSI 30 conductivity meter (YSI Inc., Yellow Springs, OH, USA) was used for conductivity measurements. A DR1900 portable spectrophotometer (Hach Company, Loveland, CO), was used to measure Fe^2+^, sulfide, silica, nitrate, and ammonia in overlying water on site. Cation concentration and trace element concentration were measured from 0.2-μm-filtered water as previously described (71–73).

Total DNA was extracted from triplicate ~250-mg samples using a DNeasy PowerSoil kit (Qiagen, Carlsbad, CA, USA). Triplicate DNA samples were pooled and sequenced on an Illumina HiSeq 2500 platform at the University of Minnesota Genomics Center (UMGC). Sickle (v. 1.33) was used to trim reads using a PHRED score of >20 and a minimum length of >50 as criteria. *De novo* assembly of short reads was performed on a per-sample basis using metaSPAdes (within SPAdes v. 3.11.0) for all samples except SM1 and SM2, which, due to high short-read yield and computational limitations, were assembled using the Ray assembler (v. 2.3.1). Contigs with sequence lengths of >2,500 bp were binned into MAGs using MetaBAT2 (v. 2.12.1). Unless otherwise noted, all bioinformatic tools were run using default parameters.

### MAG quality assessment, refinement, and taxonomic abundance estimation.

The CheckM (v. 1.1.3) lineage workflow (46) was used to assess completeness, contamination, and strain heterogeneity of all MAGs collected via binning. For MAGs with more than 10% contamination, manual coverage-based refinement was performed. Briefly, contigs with low coverage were removed, or in cases where two distinct clusters of contigs formed when GC and coverage were plotted, bins were split into separate MAGs by coverage. Refined bins were reassessed with CheckM.

Taxonomic classification was determined by pplacer (74) and/or fastANI (75) within GTDB-Tk (v. 1.3.0) (35), using the classify workflow and database version 89. The classify workflow used an aligned set of 120 concatenated bacterial single-copy marker genes (bac120) (35) from the 221 MAGs introduced in this study and 45,776 GTDB genomes. The percent abundances of recovered MAGs were estimated using read mapping with BBMap (v. 38.82) (76), adapted from an RPKG (reads per kilobase of genome per gigabase of metagenome) method described previously (50, 77). Briefly the percentage of sample reads mapped to each MAG was normalized to genome size, then divided by the sum of normalized read abundances, and multiplied by the ratio of mapped reads to total reads within a sample.

The formula used to calculate percent abundances was as follows:

$$\frac{\%\;\text{of total reads mapped to MAG}}{\text{MAG size}\left(\text{bp}\right)}÷\sum \frac{\;\text{\% of total reads mapped to MAG}}{\text{MAG size}\left(\text{bp}\right)}\times \frac{\text{total mapped reads}}{\text{total sample reads}}$$

or, briefly,

$$\text{\% abundance=}\frac{\text{normalized \% read abundance of MAG}}{\text{total normalized \% read abundances}}\times \;\text{\% sample reads mapped}$$

The results of this method are directly proportional to RPKG, except that they sum to 100. This was chosen because our conclusions were primarily drawn from intrasample relative abundances. Furthermore, low overall RPKG values in some samples compared to others limited the ability to interpret intrasample relative abundance. For comparison, an RPKG plot corresponding to Fig. 2 can be found in Fig. S3.

10.1128/msystems.00317-22.3 FIG S3 RPKG by temperature. RPKG of all MAGs with >2% relative abundance in at least one sample. Unmapped reads, low quality bins, and low abundance MAGs are grouped per sample. Download FIG S3, TIF file, 1.3 MB. Copyright © 2022 Kees et al. 2022 Kees et al. https://creativecommons.org/licenses/by/4.0/ This content is distributed under the terms of the Creative Commons Attribution 4.0 International license.

### Phylogenomic analysis.

From the 45,997 total aligned and concatenated bac120 sequences provided by the GTDB-Tk classify workflow, a selected set pertaining to 38 cyanobacterial MAGs and 79 reference genomes (74 RefSeq and 5 GenBank genomes) was extracted. Reference genomes included those related to the 38 cyanobacterial MAGs (relatedness determined by GTDB-Tk) along with a set of outgroups. A phylogenomic tree was constructed from selected alignments using RAxML (v. 8.2.11; 1,000 bootstraps, protein gamma model automatically selected) (78) and rooted by the midpoint (Fig. S1).

### Pangenome analysis and functional annotation.

Pangenomes for MAGs predicted as A/B-like *Synechococcus* clades were constructed, visualized, and analyzed using the anvi’o pangenomics workflow as previously described (79), including closely related reference genomes in the analysis where possible. Gene clusters were predicted by first calculating open reading frame similarities using BLASTp (80) and then using the MCL algorithm (81) to identify clusters within the BLASTp results. The inflation parameter for MCL was set to 10, to allow for high sensitivity in gene clustering across closely related genomes and decrease the occurrence of paralogs within clusters. Hierarchical clustering of gene clusters was performed using Euclidean distance and Ward linkages, and gene clusters were ordered in pangenome visualizations according to frequency. The BLASTp algorithm (80) was used to predict clusters of orthologous groups (COGs) by searching against the 2020 NCBI COG database (82). A second set of annotations, including EggNOG (v5 database) (83), KEGG pathways/modules/orthologs (84), gene ontology (GO) terms (85), and CAZy terms (86), were all provided with eggNOG-mapper (v. 2.0.1) (87). A set of 71 bacterial single-copy marker genes was extracted from MAGs and reference genomes in the *Synechococcus*-like pangenome, concatenated and aligned within anvi’o, and used to construct a maximum-likelihood tree using with RAxML (100 bootstraps; PROTGAMMA variable set to auto). MAGs were then ordered by the maximum-likelihood tree for displaying the pangenome, including average nucleotide identity heat maps constructed using pyANI (default ANIb, using blast+ for alignments) (88) within anvi’o (48). MAGs were grouped into taxonomic clusters within anvi’o, which were then used to calculate enrichment of gene clusters with predicted functions.

### Functional homogeneity analysis.

Gene cluster summaries were exported from the *Synechococcus*-like pangenome, which included aligned sequences, annotations, paralogs per genome, and function/geometric homogeneity scores. Gene clusters were then filtered in R by setting the maximum number of paralogs to 1, maximum functional homogeneity to 0.85, and minimum geometric homogeneity to be greater than functional homogeneity. Sequences from the resulting filtered gene clusters were extracted from the pangenome and aligned on a per-cluster basis using Clustal Omega (89). Each alignment was used to construct a distance matrix and tree using RAxML (78) and then used to generate a heat map with R. Gene clusters that were diverged in the A′ clade from the other clades were then selected by curation, using distance heat maps as a guide.

### Data availability.

Metagenome-assembled genomes have been deposited under NCBI BioProject no. PRJNA807728 and BioSample no. SAMN26200794 to SAMN26200809. Full metagenome assemblies have been deposited in the Joint Genome Institute Integrated Microbial Genomes and Microbiomes database under the accession numbers listed in Table 1.
