# Supplementary figures and images for: Distribution and Genomic Variation of Thermophilic Cyanobacteria in Diverse Microbial Mats at the Upper Temperature Limits of Photosynthesis
Source: mSystems. 2022 Aug 18;7(5):e00317-22. doi: 10.1128/msystems.00317-22 (PMC9600594; doi:10.1128/msystems.00317-22)

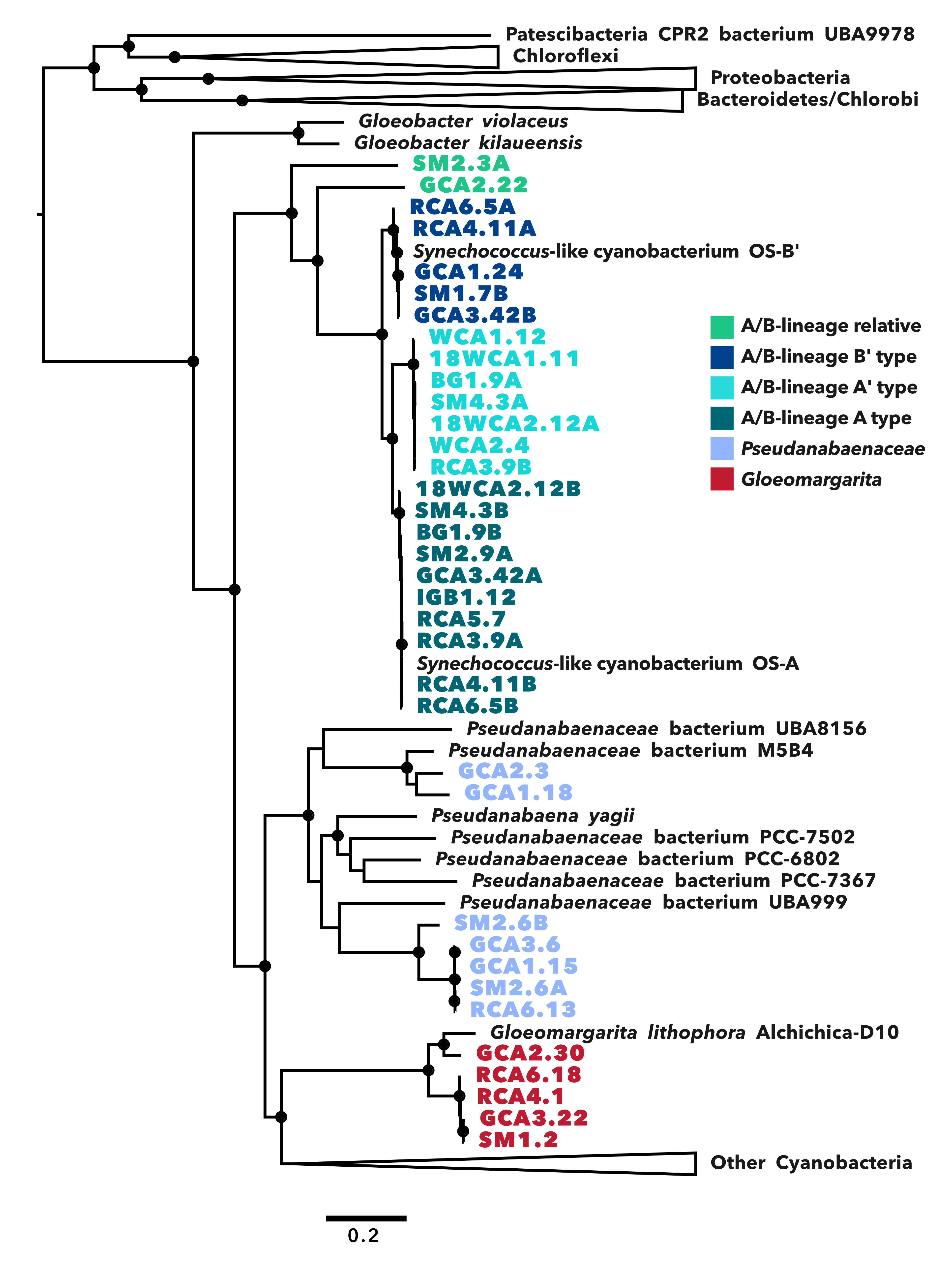

Supplement: FIG S1 [file msystems.00317-22-s0005.tif]

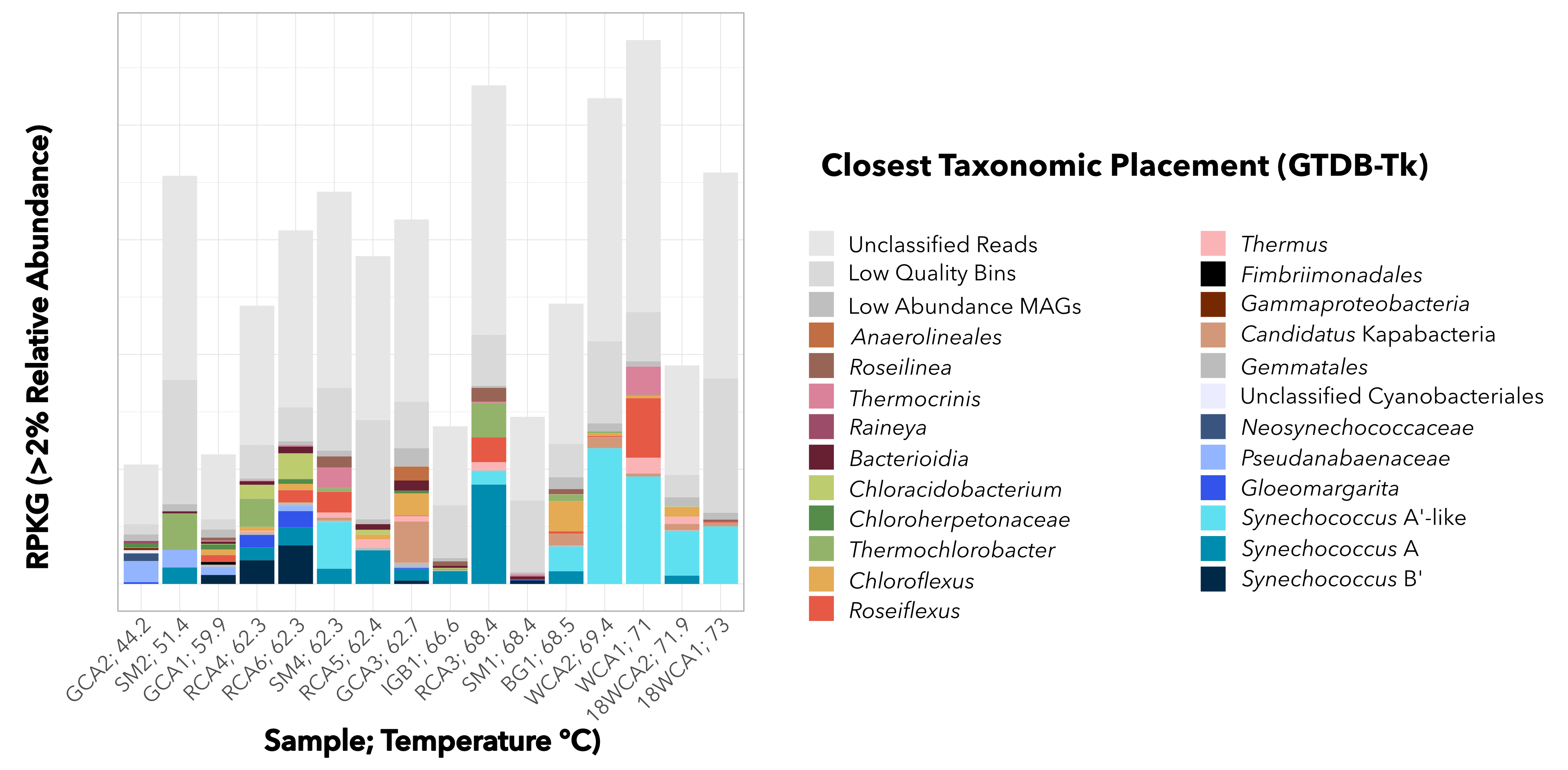

Supplement: FIG S2 [file msystems.00317-22-s0006.tif]

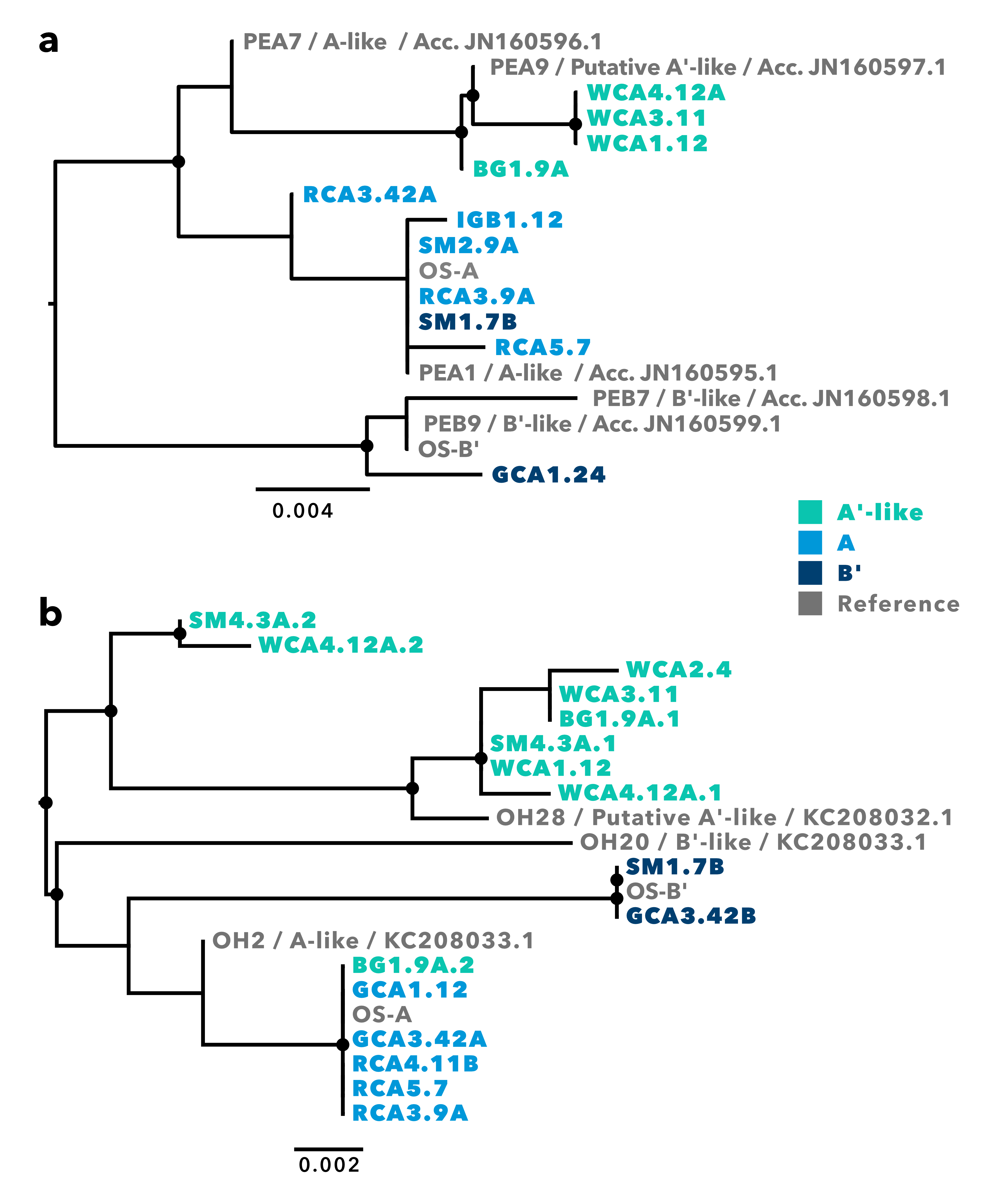

Supplement: FIG S3 [file msystems.00317-22-s0007.tif]
