# Supplementary material for: Distribution and Genomic Variation of Thermophilic Cyanobacteria in Diverse Microbial Mats at the Upper Temperature Limits of Photosynthesis
Source: mSystems. 2022 Aug 18;7(5):e00317-22. doi: 10.1128/msystems.00317-22 (PMC9600594; doi:10.1128/msystems.00317-22)
Supplement: TABLE S1 [file msystems.00317-22-s0008.docx]

Table S1. Nitrogen and protochlorophyllide reductase proteins encoded in A/B-lineage pangenome (15 MAGs, 2 reference genomes)

| Gene Cluster | BLASTP hits; predicted protein/function | Number of genomes present | | |
| --- | --- | --- | --- | --- |
|  |  | A'-clade (N=6) | A-clade (N=7) | B’-clade (N=4) |
| GC_00001963 | ChlB; Dark-operative Pchlide reductase subunit B | - | 7 | 4 |
| GC_00001829 | ChlN; Dark-operative Pchlide reductase subunit N | - | 7 | 4 |
| GC_00001904 | ChlL; Dark-operative Pchlide reductase subunit L | - | 7 | 4 |
| GC_00000650 | LPOR; Light-dependent Pchlide oxidoreductase | 6 | 7 | 4 |
| GC_00002189 | NifH; Nitrogenase iron proten | - | 3 | 4 |
| GC_00002250 | NifK; Nitrogenase molybdenum-iron protein beta chain | - | 3 | 4 |
| GC_00002252 | NifD; Nitrogenase molybdenum-iron protein alpha chain | - | 3 | 4 |
| GC_00002358 | NifN; Nitrogenase iron-molybdenum cofactor biosynthesis protein | - | 3 | 4 |
| GC_00002378 | NifE; Nitrogenase iron-molybdenum cofactor biosynthesis protein | - | 3 | 4 |
| GC_00002461 | FdxB; nif-related ferredoxin III | - | 3 | 3 |
| GC_00002441 | NifS; Cysteine desulfurase; nitrogenase Fe-S cluster biosynthesis | - | 3 | 3 |
| GC_00002363 | NifU; nitrogenase Fe-S cluster biosynthesis protein | - | 3 | 3 |
| GC_00002182 | NifV; Homocitrate synthase | - | 3 | 4 |
| GC_00002239 | NifW; Nitrogenase accessory protein | - | 3 | 4 |
| GC_00002280 | NifZ; Nitrogenase accessory protein | - | 3 | 3 |
| GC_00002494 | OS-B’ Cluster 2 UreA; Urease gamma subunit^a^ | - | 1* | 4 |
| GC_00002495 | OS-B’ Cluster 2 UreB; Urease beta subunit^a^ | - | 1* | 4 |
| GC_00001946 | OS-A/B’ Cluster 2 UreD; Urease accessory protein^a^ | - | 7 | 4 |
| GC_00002701 | UreC; Urease alpha subunit | - | 1 | 3 |
| GC_00002300 | UreA; Urease gamma subunit | - | 7 | - |
| GC_00002330 | Cluster 2 UreB2; Urease beta subunit | - | 7 | - |
| GC_00003234 | UreF; Urease Accessory protein | - | 1 | - |
| GC_00000263 | NtcA; Global Nitrogen regulator | 6 | 7 | 4 |
| GC_00001480 | Amt; Ammonium transporter | 4 | 5 | 4 |
| GC_00002094 | NirA; Ferredoxin-nitrite reductase^b^ | 5 | 2 | 3 |
| GC_00001959 | NarB; Ferredoxin-nitrate reductase | 5 | 3 | 1 |
| GC_00002096 | NrtA; Nitrate binding protein^b^ | 5 | 2 | 3 |
| GC_00002504 | NrtB; Nitrate ABC transporter permease^b^ | 4 | - | - |
| GC_00002505 | Nitrate ABC transporter permease^b^ | - | 2 | 3 |
| GC_00002344 | NrtB; Nitrate ABC transporter permease^b^ | 2 | 2 | 3 |
| GC_00001785 | NrtC; Nitrate ABC transporter ATP-binding protein^b^ | 5 | 2 | 4 |
| GC_00000342 | NrtD; Nitrate ABC transporter ATP-binding protein^b^ | 3 | 6 | 4 |
| GC_00001797 | Cons. Nitrate reductase associated protein^b^ | 5 | 3 | 2 |

^a^ – Annotation based on Bhaya et al, 2007.

^b^ – Annotation based on blastp search of *Synechococcus elongatus* PCC 7942 / FACHB-805 gene against A'/A/B’ pangenome gene clusters, then forward blastp search against refseq database.
