# Supplementary material for: Distribution and Genomic Variation of Thermophilic Cyanobacteria in Diverse Microbial Mats at the Upper Temperature Limits of Photosynthesis
Source: mSystems. 2022 Aug 18;7(5):e00317-22. doi: 10.1128/msystems.00317-22 (PMC9600594; doi:10.1128/msystems.00317-22)
Supplement: TABLE S2 [file msystems.00317-22-s0009.docx]

| Protein encoded | | | |
| --- | --- | --- | --- |
| ADK | RecO_C | Ribosomal_L29 | Ribosomal_S6 |
| AICARFT_IMPCHas | Ribonuclease_P | Ribosomal_L3 | Ribosomal_S7 |
| ATP-synt | Ribosom_S12_S23 | Ribosomal_L32p | Ribosomal_S8 |
| ATP-synt_A | Ribosomal_L1 | Ribosomal_L35p | Ribosomal_S9 |
| Adenylsucc_synt | Ribosomal_L13 | Ribosomal_L4 | RsfS |
| Chorismate_synt | Ribosomal_L14 | Ribosomal_L5 | RuvX |
| EF_TS | Ribosomal_L16 | Ribosomal_L6 | SecE |
| Exonuc_VII_L | Ribosomal_L17 | Ribosomal_L9_C | SecG |
| GrpE | Ribosomal_L18p | Ribosomal_S10 | SecY |
| Ham1p_like | Ribosomal_L19 | Ribosomal_S11 | SmpB |
| IPPT | Ribosomal_L2 | Ribosomal_S13 | TsaE |
| OSCP | Ribosomal_L20 | Ribosomal_S15 | UPF0054 |
| PGK | Ribosomal_L21p | Ribosomal_S16 | YajC |
| Pept_tRNA_hydro | Ribosomal_L22 | Ribosomal_S17 | eIF-1a |
| RBFA | Ribosomal_L23 | Ribosomal_S19 | ribosomal_L24 |
| RNA_pol_L | Ribosomal_L27 | Ribosomal_S2 | tRNA-synt_1d |
| RNA_pol_Rpb6 | Ribosomal_L27A | Ribosomal_S20p | tRNA_m1G_MT |
| RRF | Ribosomal_L28 | Ribosomal_S3_C |  |
